# Retrospective evaluation of the oral brush biopsy in daily dental routine — an effective way of early cancer detection

**DOI:** 10.1007/s00784-022-04620-9

**Published:** 2022-07-26

**Authors:** Felix W. Neumann, Heinrich Neumann, Sybille Spieth, Torsten W. Remmerbach

**Affiliations:** 1 Department of Oral and Maxillofacial and Facial Plastic Surgery, Section of Clinical and Experimental Oral Medicine, University Hospital Leipzig, Liebigstraße 10-14, 04103 Leipzig, Germany; 2 Institute of Histology, Cytology and Molecular Diagnostics Bonn (MVZ), Am Propsthof 3, 53121 Bonn, Germany

**Keywords:** Brush biopsy, Liquid-based cytology, Oral cancer, Squamous cell carcinoma

## Abstract

**Objectives:**

Oral brush biopsies are a well researched index for early detection of oral cancer in specialised centers. But the performance of the exfoliative biopsy is not yet researched in daily dental routine.

**Methods:**

Private dentists and private oral surgeons in Germany took brush biopsies out of 814 suspicious lesions from 670 patients using the Orcellex brush while regular dental appointments. The analyses of the biopsies were performed by the Cytological Laboratory of Bonn (CLB) using liquid-based cytology.

**Results:**

The final results were 74 oral squamous cell carcinomas and one verrucous carcinoma, histological proven, 232 cases of leukoplakia, 242 cases of lichen planus, 17 cases of erythroplakia, 259 cases of benign inflammatory, traumatic or hyperplastic oral lesions. The sensitivity for the detection of cancer cells using brush biopsy archived 100%, the specificity for the detection of non-neoplastic cells was 86.5%. The positive predictive value was 43.1%, the negative predicative value was at 100%.

**Conclusion:**

The oral brush biopsy seems to be a sufficient tool for early cancer detection in private dental offices.

**Clinical Relevance.:**

Generally, practicing dentists do not see various oral squamous cell carcinomas in their careers, so the experience in identifying oral squamous cell carcinomas as such is very low. The brush biopsy might help them in cases of doubt to prevent tumors from expansive growth.

## Introduction

Cancer is one of the main causes of death worldwide. In Germany alone, 14,310 new cases of oral cancer and cancer of the throat were found in 2018. The survival rate of the patients suffering from oral SCC is very poor. Only around 50% survive the first 5 years after diagnosis [1]. The low survival rate of oral SCC is often based on the late stage of first diagnosis of the cancer [2]. It has been shown that the stage of the cancer at time of diagnosis is the main factor for a better survival of the patient. Only 16.2–32% of the patients with a stage 4 oral SCC survive the first 5 years, contrasted with approximately 80% of the patients with a stage 1 oral SCC [2, 3]. This clearly shows the importance of developing and establish early detection methods of abnormal cell growth. We argue that especially methods that are standardised, easy to perform and accurate have the potential to save many lives.

One technique that could be able to fulfill all these expectations is the oral brush biopsy, an exfoliative biopsy of the oral mucosa [3]. It is minimally invasive compared to the excision, easy to perform, and it is efficient in collecting representative cells of the mucosal lesion [3, 4]. Furthermore, it is usually completely painless. That is why most patients show a high acceptance for this technique [3, 5].

The oral brush biopsy is a well-researched topic. Many studies deal with the sensitivity and specificity of brush biopsy in the environment of a university hospital and in specialized tumor centers [3–7, 15–30]. The latest meta analysis of the Cochrane Library with the topic of diagnostic tests for oral cancer and potentially malignant disorders reported about the efficiency of the methods vital staining, light-based detection and oral cytology. They included over 60 studies, of which 24 examined oral cytology. Most of these used oral brush biopsies for harvesting cells from the oral mucosa. In the discussion, the authors of the Cochrane review argue that the promising results of a combined sensitivity of 90% and a combined specificity of 94% for the oral cytology cannot be directly applied to daily dental routine. This is due to the fact that no studies yet exist that explore results of “frontline clinicians.” The authors say it could be possible that “*more false positives would be generated leading to unnecessary referrals, more false negatives could also be the case if tests were not performed correctly, and it is also possible that the tests would be robust enough to be the same in general and specialist practice*” [8].

The German health care system covers the examination of suspicious oral lesion using oral brush biopsy since 2004. The German guideline of treatment and detection of pre-malignant oral lesions also found strong consent for the use of oral brush biopsies for a cytological analysis in cases where the dentist is not sure of the presence of a tumor [9]. This provides the perfect conditions to meet the Cochrane Review’s requirement.

This study used the Data gained by regular dental appointments in generally practicing dental offices and private oral surgeons in Germany to examine the efficiency of the oral brush biopsy in primary care. It presents the results of a single center study analysis of pseudonymised data collected by the cytologic laboratory in Bonn from April 2014 until December 2016.

The aim of this study is to examine if the brush biopsy of the oral mucosa is a sufficient tool for early cancer diagnosis in general dental routine, performed by general dentists in primary care, not only by specialised surgeons.

## Material and methods

The data used in this study was generated by the CLB, a commercial cytologic and pathologic laboratory in Bonn, Germany. Therefore, private dental clinics and private oral surgeons took oral brush biopsies from suspicious lesions at the oral mucosa of their patients. The inspection of the mucosa was part of the regular dental check-up. The results were used pseudonymised without trespassing personal ID-Information but the age and gender of the patients.

**Fig. 1:**
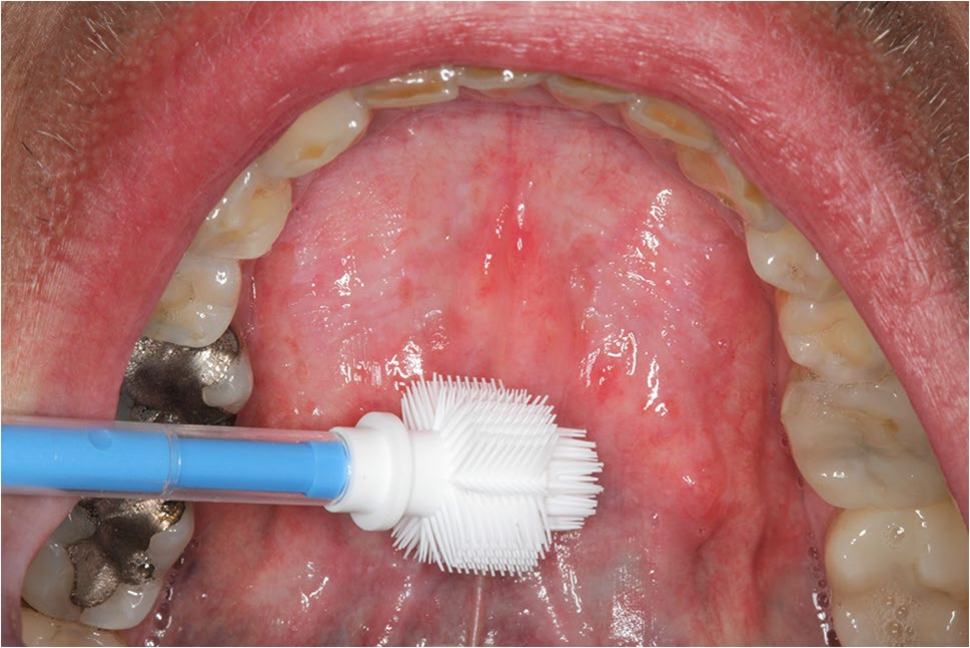
Positive for tumor cells: There are huge differences in size, form and staining of the nuclei together (1) with an increased Nuclear/cytoplasmatic (N/C) ratio (2). There is proteinaceous necrotic debris, i.e. tumor diathesis (3). Dark irregular groups of highly atypical cells (4) and signs of abnormal keratinisation of some tumor cells (5). These abnormal findings are visible in all fields of view. Picture by H. Neumann

**Fig. 2:**
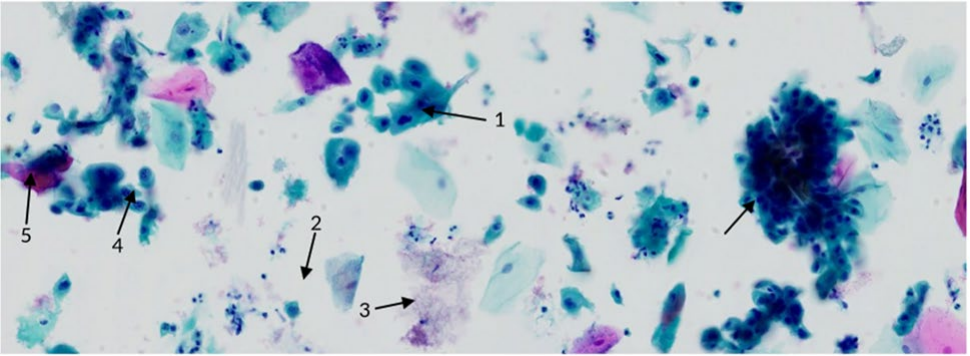
Suspicious for tumor cells: The high nuclear atypia can be appreciated in those nuclei that are in focus. (1) Uneven nuclear contour with several nuclei. (2) Opaque nucleoli, representing atypical pykosis. (3) Atypical mitotic figure. In this case, this was the only cell group with these characteristics. Picture by H. Neumann

**Fig. 3:**
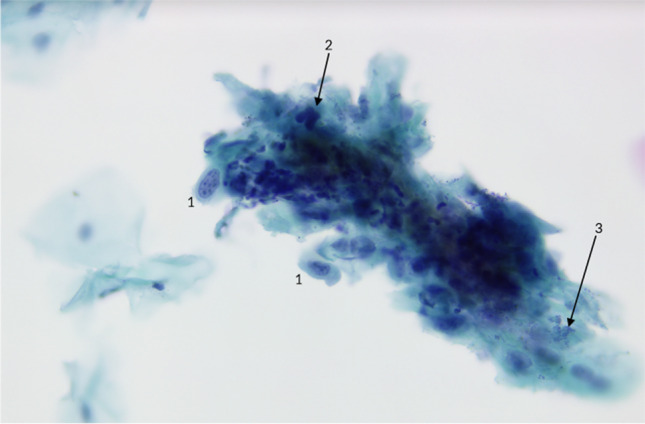
Doubtful: (1) numerous cells with small perinuclear clearing (“halos”). (2) mild differences in size, form and staining of the nucleoli. (3) Polymorphis infiltrating groups of squamous epithelia. (4) branching and septated hyphae of *Candida albicans*. Picture by H. Neumann

**Fig. 4:**
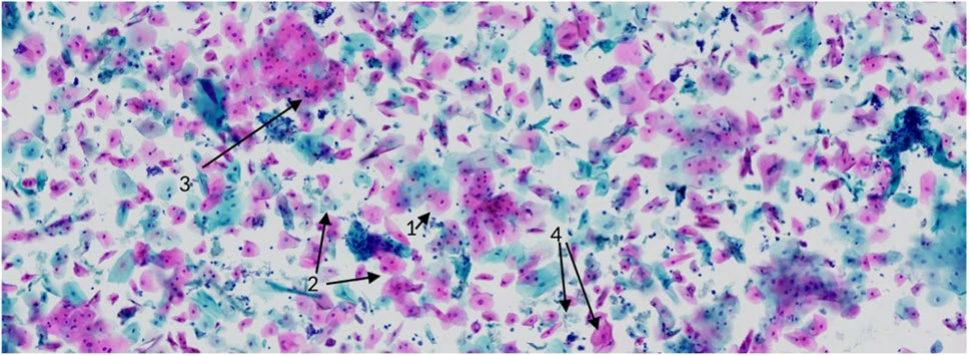
Negative for tumor cells: Clear background, normal squamous cells, small nuclei, low N/C ratio. Minimal variation in nuclear form. Picture by H. Neumann

**Fig. 5:**
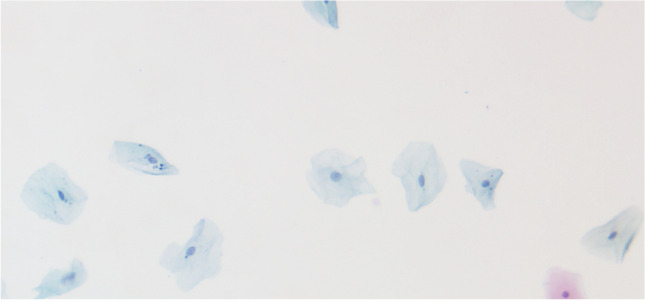
Homogeneous Leukoplakia on the left and right side of the anterior part of floor of the mouth. Additionally a current version of an Orcellex cell collector is presented. Picture by T.W. Remmerbach

Generally, the samples were collected using the Orcellex® brush (Rovers Medical Devices B.V. Oss, The Netherlands) between April 2014 and December 2016 (Fig. 1) and analyzed using liquid-based cytology. For collecting cells out of the superficial and middle layer of the mucosa, the brush was placed at the doubtful lesion and twisted ten times with moderate pressure. The head of the brush was immediately transferred into a preservation fluid, in all cases the BD SurePath™ Collection Vial (BD Diagnostics, TriPath, Erembodegem, Belgium), to preserve the obtained cells. All cytological diagnostics were performed at the Cytologic Laboratory of Bonn (CBL). First the cells were extracted out of the liquid. Then, preparation and staining with the Papanicolaou-protocol was performed automatically using the PrepMate system (Becton Dickinson (AutoCyte), Franklin Lakes, New Jersey, USA). During this process, the cells were selected using sedimentation and most of the inflammatory cells and other diagnostically irrelevant parts of the sample were removed. The final sample was placed in a thin layer between a microscope slide and a cover slip, within a circle of a diameter of 13 mm and examined by experienced cytopathologists (HN, SS). The diagnoses were classified as “negative,” “suspicious,” “doubtful,” or “positive” for tumor cells, or as “insufficient for proper analysis” [16] (Figs. 2, 3, 4, 5). A “positive” result means at least one malignant tumor cell was found in the sample; “suspicious” samples contained cells that have had at least moderate signs of malign transformation. A “doubtful” sample contained cells that were mildly transformed and in a “negative” sample, no abnormalities were found.

Only cytological diagnoses that were “negative” were counted as overall negative. All diagnoses classified as “suspicious,” “doubtful” or “positive” were counted as overall positive diagnoses and further investigations were performed, such as histological biopsies. The lesions with a negative result were periodically controlled by clinical follow-up appointments.

Because all used data have been pseudonymised before the usage in this study, no ID-information but gender and sex were used and the data was gained in regular dental check-up surroundings, not especially for developing a scientific study, the ethical committee of Nord-Rhein Germany did not see the necessity of a separate ethical approval. We respected the guidelines of the Helsinki Declaration.

## Results

From April 2014 to December 2016, a total of 814 brush biopsies were collected from the same number of suspicious lesions from 670 Patients. Three biopsies (= 0.3%) were technically insufficient and were excluded from further analysis. Further three of the patients were under the age of 18 and were also excluded of the further analysis.

The biopsies were taken by 40 dentists in private dental offices, four of these were specialised for oral surgery and/or maxillofacial surgeons. Most of the dentists took between 1 and 20 samples in the respective observation period, surgeons obtained between 11 and 100 brush biopsies. The reason for taking samples were suspicious lesions of the oral mucosa, the lip and the tongue. We compared the results of the brush biopsies with the results of the histological analysis of the same lesion or a clinical follow-up, respectively.

The dentists were following the treatment and diagnostic guidelines for potentially malignant disorders of the Association of the Scientific Medical Societies in Germany [9]. Therefore, lesions with urgent clinical signs of malignancy were examined by a surgical biopsy. At other lesions of the mucosa that were doubtful of malignity an oral brush biopsy was performed. The dentists had not undergone a special training for cancer diagnosis but were general practitioners of the oral health care system in Germany.

In summary, 47% of the patients were men (315 cases) with a mean age of 59.0 years and 53% were women with a mean age of 64.1 years. Thus, women were on average 5.1 years older than men (Table 1). The age of all patients varied from 20 up to 96 years of age. The reasons for the brush biopsies were the suspicions of oral SCC or its recurrence (10.4% together), control of leukoplakia (30.2%) or lichen (27.6%). The rest of the biopsies were taken at mechanically damaged or inflamed tissue that was resistant to treatment (Table 2). The suspected diagnoses of the patients diagnosed with a malignant tumor were mostly oral SCCs (65.4%), lichen (14.6%) or non-healing mechanical irritations (9.3%) (Tables 3 and 4). The final diagnoses in this study were 74 cases of oral SSC and one case of oral verrucous SSC (= 9.2%), 232 cases of leukoplakia, 242 cases of lichen planus, 17 cases of erythroplakia, 242 cases of benign inflammatory, traumatic, or hyperplastic oral lesions (Table 5). 61 of the oral SCCs were found in men (= 81.3%) with an average age of 75 years. The other 14 oral SCCs were found in women with an average age of 68 years. The youngest patient with a detected oral SCC was 37 years old, the oldest 90. Given that we almost had a 50:50 ratio of male to female patients, this means that of the patients analyzed in this study, men were four times more likely to develop oral cancer than women.

**Table 1:** Results of the brush biopsies and average age

Result brush biopsies	Sample size and average age (years)		
	Male	Female	Total
Positive	45	16	61
	58.9	68.0	61.3
Suspicious	15	16	31
	65.0	70.0	67.5
Doubtful	36	45	81
	58.7	67.8	63.8
Negative	284	351	635
	58.8	63.2	61.2
Total	380	428	808
	58.9	64.0	61.6

**Table 2:** Suspected diagnosis as suggested using brush biopsy and cytological analysis

Clinical diagnosis	Frequency	
	Absolute	Relative
Oral SCC	83	10.3%
Leukoplakia	242	29.9%
Erythroplakia	22	2.7%
Lichen	223	27.6%
Other reasons	238	29.4%
Total	808

**Table 3:** Cytological results of patients with suspected diagnosis: “oral SCC”

Result brush biopsies	Final diagnosis (positive for oSCC or negative for oSCC)		
	Tumor positive	Tumor negative	Total
Positive	30	0	30
Suspicious	1	1	2
Doubtful	2	3	5
Negative	0	46	46
Total	33	50	83

**Table 4:** Cytological results of patients with suspected diagnosis: “oral SCC”

Result brush biopsies	Final diagnosis (positive for oSCC or negative for oSCC)		
	Tumor positive	Tumor negative	Total
Positive	30	0	30
Suspicious	1	1	2
Doubtful	2	3	5
Negative	0	46	46
Total	33	50	83

**Table 5:** Final proven diagnosis all patients

Result brush biopsies	Final diagnosis (positive for oSCC or negative for oSCC)		
	Tumor positive	Tumor negative	Total
Positive	26	5	31
Suspicious	10	19	29
Doubtful	6	70	76
Negative	0	589	589
Total	42	683	725

In this study, we summarized the cytological diagnoses of “suspicious,” “doubtful,” and “positive” for malignancy as positive cytological results. This led to an overall sensitivity for the detection of cancer cells using brush biopsy of 100%; the specificity for the detection of non-neoplastic cells archived 86.5%. The positive predictive value was 43.1%, the negative predictive value was at 100% (Tables 6, 7 and 8). There were 61 positive diagnoses of the brush biopsies of which 56 were later histologically proven as oSCCs. Seven cases turned out to be false positive. Furthermore, 11 of the 31 suspicious and 8 of the 81 doubtful cytological diagnoses turned out to be oral SCCs. Of the 75 diagnosed oral SCCs, 17 had still been in the stage of a carcinoma in situ. Only 11 of the SCCs were diagnosed in the late stage of pT4 (Table 9).

**Table 6:** Diagnostic accuracy of the brush biopsy overall

Test	Accuracy
Sensitivity	100%
Specificity	86.5%
Positive predictive value	43.1%
Negative predicative value	100%

**Table 7:** The data from clinically suspected carcinomas separately had a sensitivity of 100%, a specificity of 92.0% and the positive predictive value was 89.2%

Clinically suspected carcinomas		Cytological diagnosis		Total
		Negative	Positive	
Cancer diagnosed	Positive	0	33	33
	Negative	46	4	50
Total		46	37	83

**Table 8:** The data from clinically suspected oral potentially malignant disorders had a sensitivity of 100%, a specificity of 86.2% and the positive predictive value was 30.9%. The negative predictive value was in both cases at 100%

Clinically suspected potentially malignant disorders		Cytological diagnosis		Total
		Negative	Positive	
Cancer diagnosed	Positive	0	42	42
	Negative	589	94	683
Total		589	136	725

**Table 9:** Staging parameter of tumor growth during the diagnosis

Stage of tumor growth	Frequency	
	Absolute	Relative
Carcinoma in situ	17	22.6%
pT1	15	20.0%
pT2	20	26.6%
pT3	12	16.0%
pT4	11	14.6%
Total	75	

## Discussion

The results of this study show a relative high number of negative diagnoses and a small proportion of positive cases. This might be because the general practitioners have less experience in the clinical diagnosis of malignant tumors due to low prevalence of oral cancer. The lack of knowledge about oral cancer of German dentists is a problem that can be dealt with through training and lecture [11]. Clinical cases of potentially malignant disorders show an obvious higher prevalence, but the clinical experiences of dentists in Oral Medicine is still low and the uncertainty how to deal with such lesions is widespread. An experienced surgeon of a specialized center might be more sovereign in visual diagnosis of cancer and the classification of an oral lesion than a general dentist. In the clinical inspection, the specialized surgeons are better at selecting which lesions require a brush biopsy and which do not. The brush biopsy is a suitable tool for general dentists to compensate this lack of experience. It seems to be robust against obtainment (geographic) errors while being performed since only three brush biopsies (= 0.3%) were insufficient for cytological diagnosis.

The fact that this technique is not frequently used in German dentistry — although it is covered by the German health insurance system and mentioned in the treatment guidelines [9] — could be due to a lack of knowledge of the dentists or too hastily performed examination of the mucosa in the regular dental check-up [11]. Both are possible errors that should be examined in further studies. In Florida, only 25% of patients suffering from oSCC had a sufficient examination of the oral mucosa at their last dental check-up [12]. Only half of the dentists in a German study said that their knowledge about oral cancer is sufficient and only 28% performed a detailed examination of the oral mucosa of all their elderly patients. Through one educational meeting after a period of 1 year, this proportion could have been raised to 37% through further education of these dentists [11]. Thus, it seems that repeated sensitization of the dentists could improve the awareness and the diligence for this topic.

The stage of the tumors while detection appears to be, compared to the average stage of tumors of the oral cavity and the throat in Germany, relatively low. The German Robert-Koch-Institute (RKI) has reported that the cancer of the oral cavity and the throat in the years of 2017 and 2018 were diagnosed in stage 1 in about 24%, stage 2 in 17%, stage 3 in 17%, and stage 4 in 42% with male patients and that female patients were diagnosed with a tumor at stage 1 in 32%, stage 2 at 15%, stage 3 at 17%, and stage 4 at 36% of all cases [1].

The results of this study can be easily compared with those of the RKI, as these provide an overview of all cancers in Germany and might as such be the best comparison group. Our study has shown a cancer stage at diagnosis of 42% in tumor stage 1 and carcinoma in situ; 26.6% in tumor stage 2, 16% in tumor stage 3, and 14.6% in tumor stage 4. Thus, the part of early stage tumors (stage 1 and 2 in sum) were at 68.6% in our study compared to 47% of the women and 41% of the men included in the data of the RKI. It seems that at least 21% of the patients received an earlier diagnosis than average by using oral brush biopsies.

The preferred German diagnostic scheme of extragenital cytology is, as mentioned above, organized in four categories [10]. Nevertheless, the clinical consequence of different interpretation algorithms needs to be discussed. In this study, we defined all non-negative cytological results to be “positive”. Some studies, however, combine negative and suspicious diagnoses to be negative and take only doubtful and positive diagnoses as “positive”. Thus, we have applied their methodology to allow for better comparability. When applying this different categorization, our sensitivity changes to 86.7% and the specificity is 96.3%.

For clinical use, costs and benefits need to be assessed. If a patient’s cancer is not detected by the oral brush biopsy through a false-negative result, the tumor may continue to grow and put the patient’s life at serious risk. On the other hand, if a patient is falsely positively diagnosed, an unnecessary scalpel biopsy might be performed. The psychological impact of a false-positive diagnosis on the patient is not to be underestimated. Still, the benefit of one correct positive result is arguably much greater than the damage done by one false-positive one, especially, if one follows the recommendation of Lingen et al. (2017) to perform a scalpel biopsy in every case of suspicious lesion [13]. For the non-specialised and generally practicing dentist, a lower specificity should generally be tolerated in exchange for a better sensitivity rate. The resulting increased rate of false positive results should be accepted.

Some authors demand that every oral cytological diagnosis should be confirmed or refuted by a histological analysis. They further suggest that it would be best if the excision would be performed in the same appointment as the oral brush biopsy [14]. The data we analyzed were surveyed in the clinical practice to see whether this technique is appropriate for frontline clinicians. The fact that the dentists took the brush biopsy as a first diagnostic tool of a mucosal lesion makes this demand impracticable for this type of study.

The sensitivity and specificity found in the Cochrane meta-analysis for oral cytology are at a sensitivity of 90% and a specificity of 94% [8]. This includes studies that collected cells by a metal spatula as well as studies that made conventional cytology and liquid-based cytology. The conventional cytology paired with the liquid-based cytology alone gained a pooled sensitivity and specificity of 93.88% and 90.36% out of biopsies from 2601 patients [15–30].

The studies of liquid-based cytology of oral lesions included in the Cochrane review gained a pooled sensitivity and specificity of 83.92% and 97.55% out of biopsies from 677 patients [15–21]. Three of these worked with DNA image cytometry in addition to the oral brush biopsy as an adjuvant analysis [17, 18, 21].

All these studies but one worked with data out of a specialised center or the university clinic. For the other study, specialists went into the province of India and performed the cytology with a mobile laboratory [17]. None of these studies worked with general dentists as the performing therapist.

The Cochrane review notes that the effectiveness of the oral brush biopsy has not yet been researched in the frontline. This study suggests that oral brush biopsy is a good tool for early cancer detection in dental offices. It seems to be a good method for practitioners who are inexperienced in dealing with oral premalignant and malignant oral lesions to gain certainty in the diagnosis and to ensure early tumor detection, too. The technique seems to be robust enough to be carried out safely even by inexperienced practitioners.

## Conclusion

The results of this study show that the oral brush biopsy is a useful and highly reliable tool for the early diagnostic of oral cancer in private dental practices — not just in specialised clinics. A major reason for the relatively high mortality rate of oral cancer is that it often is detected when it is too late. The main barriers of early oral cancer detection seem to be the visual examination of the oral mucosa, the education of the dentists about potentially malignant diseases and the execution of the oral brush biopsy. Oral brush biopsy is reliable enough to be an effective tool for primary oral care providers to detect oral cancer before it is too late. Especially, for general dentists that are not completely firm in dealing with oral lesions, it is a helpful tool for suspicious lesions. This might be one way to reduce cancer mortality rates that is easy, inexpensive, and reliable.
