# Correction to: Retrospective evaluation of the oral brush biopsy in daily dental routine — an effective way of early cancer detection

**DOI:** 10.1007/s00784-022-04660-1

**Published:** 2022-08-03

**Authors:** Felix W. Neumann, Heinrich Neumann, Sybille Spieth, Torsten W. Remmerbach

**Affiliations:** 1 Department of Oral and Maxillofacial and Facial Plastic Surgery, Section of Clinical and Experimental Oral Medicine, University Hospital Leipzig, Liebigstraße 10‑14, 04103 Leipzig, Germany; 2 Institute of Histology, Cytology and Molecular Diagnostics Bonn (MVZ), Am Propsthof 3, 53121 Bonn, Germany

**Correction to: Clinical Oral Investigations**

**https://doi.org/10.1007/s00784-022-04620-9**

The original version of this paper contained a transposition of Figure 1, 2, 3, 4 and Figure 5.

The original article has been corrected.

